# 5-Hydroxymethylome in Circulating Cell-free DNA as A Potential Biomarker for Non-small-cell Lung Cancer

**DOI:** 10.1016/j.gpb.2018.06.002

**Published:** 2018-07-18

**Authors:** Ji Zhang, Xiao Han, Chunchun Gao, Yurong Xing, Zheng Qi, Ruijuan Liu, Yueqin Wang, Xiaojian Zhang, Yun-Gui Yang, Xiangnan Li, Baofa Sun, Xin Tian

**Affiliations:** 1 Department of Pharmacy, The First Affiliated Hospital of Zhengzhou University, Zhengzhou 450052, China; 2 Henan Key Laboratory of Precision Clinical Pharmacy, Zhengzhou University, Zhengzhou 450052, China; 3 Sino-Danish College, University of Chinese Academy of Sciences, Beijing 100049, China; 4 Physical Examination Center, The First Affiliated Hospital of Zhengzhou University, Zhengzhou 450052, China; 5 Department of Anesthesiology, The First Affiliated Hospital of Zhengzhou University, Zhengzhou 450052, China; 6 CAS Key Laboratory of Genomic and Precision Medicine, School of Future Technology, Beijing Institute of Genomics, Chinese Academy of Sciences, Beijing 100101, China; 7 Institute of Stem Cell and Regeneration, Chinese Academy of Sciences, Beijing 100101, China; 8 Department of Thoracic Surgery, The First Affiliated Hospital of Zhengzhou University, Zhengzhou 450052, China

**Keywords:** 5-Hydroxymethylcytosine, Lung cancer, Cell-free DNA, Biomarker, 5hmC-Seal

## Abstract

Non-small-cell lung cancer (NSCLC), the most common type of **lung cancer** accounting for 85% of the cases, is often diagnosed at advanced stages owing to the lack of efficient early diagnostic tools. **5-Hydroxymethylcytosine** (5hmC) signatures in circulating **cell-free DNA** (cfDNA) that carries the cancer-specific epigenetic patterns may represent the valuable **biomarkers** for discriminating tumor and healthy individuals, and thus could be potentially useful for NSCLC diagnosis. Here, we employed a sensitive and reliable method to map genome-wide 5hmC in the cfDNA of Chinese NSCLC patients and detected a significant 5hmC gain in both the gene bodies and promoter regions in the blood samples from tumor patients compared with healthy controls. Specifically, we identified six potential biomarkers from 66 patients and 67 healthy controls (mean decrease accuracy >3.2, *P* < 3.68E−19) using machine-learning-based tumor classifiers with high accuracy. Thus, the unique signature of 5hmC in tumor patient’s cfDNA identified in our study may provide valuable information in facilitating the development of new diagnostic and therapeutic modalities for NSCLC.

## Introduction

Lung cancer is one of the most common cancers and is the leading cause of cancer-related mortality [1], [2]. In particular, non-small-cell lung cancer (NSCLC), which mainly consist of adenocarcinoma (AC, 44%) and squamous cell carcinoma (SCC, 26%), accounts for about 85% of lung cancers [3], [4]. Patients with early stages of NSCLC mostly don’t have any symptoms, leading to their diagnosis frequently at advanced stages [5]. Low-dose computed tomography (LDCT) has been used to improve the detection of early-stage lung cancer [6]. However, it is far from satisfactory as a screening approach for its low specificity and radiation risks [7], [8]. Although several recent studies have tried to discover the sensitive and specific blood-based circulating biomarkers for early detection of NSCLC using multiple omics methods, including genomics, transcriptomics, proteomics, and metabolomics, few biomarkers from clinical study have been successfully translated into clinical routine screening for lung cancer mainly due to the poor reproducibility, low sensitivity, or high false-positive rates [5], [9]. Therefore, it is potentially significant to develop highly sensitive and reliable diagnostic approaches for NSCLC.

Cell-free DNA (cfDNA) refers to the small nucleic acid fragment circulating in the plasma or serum. Tumor cells release DNA into the serum or plasma via multiple mechanisms, allowing detection of cancer-associated genetic alterations, including point mutations, copy number variations, chromosomal rearrangements, and epigenetic aberrations [10], [11]. Non-invasive biomarkers in cfDNA offer substantial advantages than tissue biopsy as they possess the entire genetic marks of tumor tissue, and their easily accessible nature makes them the ideal candidates for real-time and dynamic monitor of the treatment response [12], [13]. Detecting genetic and epigenetic biomarkers in cfDNA has emerged as a promising non-invasive approach for the diagnosis, prognosis, and treatment of cancer [12], [13], [14].

Epigenetic alterations, especially for aberrant DNA methylation processes, contribute to tumor initiation and progression [15], [16], [17]. DNA methylation, the conversion of cytosine to 5-methylcytosine (5mC), is a well-established regulator of gene expression [18]. 5-hydroxymethylcytosine (5hmC), an oxidation product of 5mC, is an intermediate product of active DNA demethylation [19]. Recent studies have shown that 5hmC plays a critical role in gene expression regulation, as well as in the carcinogenesis of multiple solid tumors [20], [21], [22]. Given its tissue- and cancer-specific distribution, DNA 5hmC may serve as an ideal biomarker for cancer diagnosis and prognosis [23]. Studies from our laboratory and others have demonstrated that the 5hmC signatures in cfDNA could serve as epigenetic biomarkers for several human cancers [24], [25], [26]. However, the potency and reliability of cell-free 5hmC as a diagnostic biomarker for NSCLC remain largely unknown.

In this study, we utilized a highly sensitive and reliable method to map the genome-wide distribution of 5hmC in the cfDNA from a cohort of 66 NSCLC patients and 67 healthy individuals. Our results revealed that 5hmC modifications in cfDNA of NSCLC patients exhibit distinct features with 5hmC gains in both gene bodies and promoters compared to those in the cfDNA of the healthy controls. Specifically, six 5hmC-based candidate biomarkers were identified in cancer patient cfDNAs. The cell-free 5hmC signatures identified in our study may provide potentially valuable biomarkers for non-invasive diagnosis of Chinese NSCLC.

## Results

### Sample characteristics and cell-free 5hmC-Seal profiling

**Figure 1:**
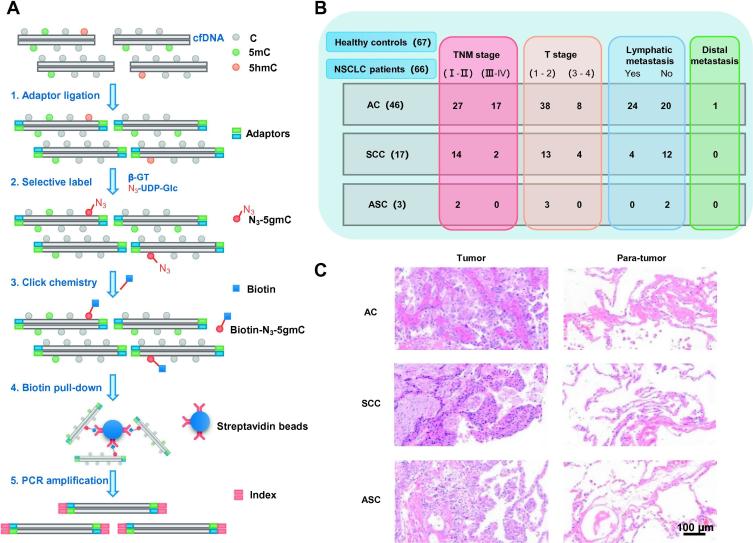
**Overview of sample information and preparation** **A.** Workflow of cell-free 5hmC-Seal-seq library preparation and sequencing. **B.** Schematic overview of clinicopathological characteristics of NSCLC patients in our cohort. **C.** Representative images of hematoxylin and eosin (HE) staining in different histological types of NSCLC and the corresponding adjacent para-tumor tissues. Scale bar, 100 µm. cfDNA, cell-free DNA; NSCLC, non-small-cell lung cancer; AC, adenocarcinoma; SCC, squamous cell carcinoma; ASC, adenosquamous carcinoma; β-GT, β-glucosyltransferase; N_3_-UDP-Glc, UDP-azide-glucose; 5-gmC, β-glucosyl-5-hydroxymethylcytosine; TNM, tumor node metastasis.

We first compared the 5hmC features of cfDNA between NSCLC and healthy individuals using a sensitive 5hmC-Seal method [27]. The 5hmC profiles in cfDNA were acquired from 66 NSCLC patients and 67 healthy controls (Figure 1A). Detailed information regarding subject characteristics, tumor features, and cancer biomarkers tested is illustrated in Figure 1B and Table S1. The average age of NSCLC patients and healthy controls was similar, which was 59 and 55 years old, respectively. The gender ratio was about 1:1 in both groups. Hematoxylin and eosin staining indicated that there were 46 AC, 17 SCC, and 3 adenosquamous carcinoma (ASC) patients in our NSCLC cohort (Figure 1B and C). Among all patients, 29% (19 out of 66) were at advanced stages (TNM stages III and IV). Moreover, 42% (28 out of 66) patients showed lymph node metastasis, and 1 patient exhibited distal metastasis (Figure 1B). As a routine test, we measured the serum levels of seven conventional cancer biomarkers, including carcinoembryonic antigen (CEA), alpha-fetoprotein (AFP), carbohydrate antigen 19-9 (CA 19-9), carbohydrate antigen 15-3 (CA15-3), carbohydrate antigen 125 (CA125), neuron-specific enolase (NSE), and cytokeratin 19 fragment (CYFRA21-1). However, positive results were only demonstrated in CEA, CA125, NSE, and CYFRA21-1 in NSCLC patients with relatively lower positive rates of 22.50%, 5.88%, 5.88%, and 37.93%, respectively (Table S1), suggesting that the traditional routine biomarkers are not sensitive enough to distinguish NSCLC patients from healthy individuals.

**Figure 2:**
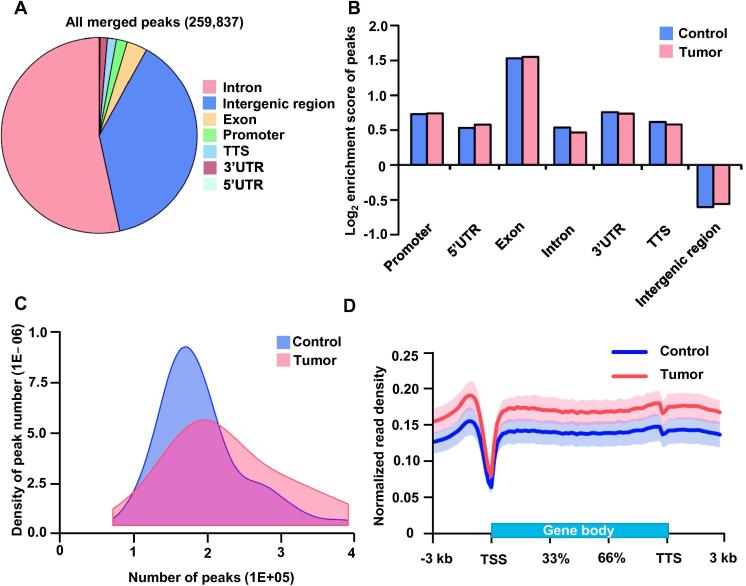
**Genome-wide distribution of 5hmC in blood samples from healthy controls and NSCLC patients** **A.** The pie chart shows the overall genomic distribution of hMRs in cfDNA. **B.** Normalized enrichment score of hMRs across distinct genomic regions relative to that expected in control and tumor samples, with positive values indicating enriched more than expected. **C.** Density distribution of peak number in blood samples from 67 healthy controls and 66 patients with NSCLC. **D.** Metagene profiles of cell-free 5hmC in healthy and lung cancer samples. Shaded area indicates the upper and lower quartile. hMR, 5hmC-enriched regions; TSS, transcription start site; TTS, transcription termination site.

To exclude the epigenetic alterations caused by gender impact, we discarded the reads located on chromosomes X and Y, and then compared the global genomic distribution of cfDNA 5hmC between tumor and control groups. The genome-wide read distribution of four samples (2 controls and 2 tumor samples, respectively) was exemplified. According to the read count, there was no obvious difference observed between the two groups (Figure S1A). To determine whether or not the cfDNA from the blood of NSCLC patients had any abnormal 5hmC enrichment in certain region, we analyzed 5hmC-enriched regions (hMRs) by HOMER and identified 259,837 hMRs in 66 lung cancer patients and 67 healthy individuals (Figure 2A). The genome-wide analysis of hMRs showed that >60% of hMRs are located in gene bodies with the highest enrichment in exons (ratio of the number of peaks observed to the number of peaks expected, o/e), whereas fewer hMRs were found in intergenic regions (Figure 2B), which is consistent with previous studies showing that the majority of 5hmC in mammals is enriched in the intragenic and promoter regions [28], [29]. Therefore, our genome-wide approach demonstrated the widely distributed and highly exon-enriched natures of cfDNA 5hmC in NSCLC patients and healthy controls.

### Heterogeneity and hyper-hydroxymethylation in NSCLC cfDNA

To further compare the difference in 5hmC features between the two groups, we calculated the density of peak number and found that the overall number of hMRs in the tumor group was higher than that of the control group with median number of 213,432 and 188,972, respectively (Figure 2C). Moreover, the tumor group exhibited broad distribution compared to the control group whose hMR distribution exhibited a narrow and sharp curve, which could be explained by the higher degree of heterogeneity in tumor tissues. In addition, we found that the overall normalized read density of cfDNA 5hmC was slightly higher in the tumor group when compared to the control samples (Figure 2D). These results illustrate a higher 5hmC enrichment in both the peak number and metagene profiles in cfDNA of NSCLC patients than that of controls (Figures 2C and S1B).

Moreover, we downloaded the public cfDNA 5hmC data retrieved from Song et al. containing 8 healthy controls, 1 corresponding input (Stanford blood center), 9 non-metastatic lung cancer and 6 metastatic lung cancer samples (West China Hospital) [25]. The average age of lung cancer patients and healthy controls for their samples were 59.5 and 61.5 years old with gender ratio of about 8:7 and 1:4 (male:female), respectively. To compare the profiles of different sample sources, we calculated normalized read density (Figure S1C) and fragments per kilobase of transcript per million fragments mapped (FPKM) of 5hmC across the whole genomic regions of all the samples. The metagene profiles of tumor groups from different sources all displayed a lower 5hmC enrichment than that of public control data, but slightly higher than our controls (Figure S1C). Hierarchical clustering analysis of all common genes present in each sample didn’t show any obviously preferred clustering for tumor groups (Figure S1D). Principle component analysis (PCA) demonstrated that the control samples from different labs are separated but well-clustered for each individual group. In contrast, all tumor groups exhibited a higher degree of heterogeneity (Figure S1E). These findings indicate a consistent 5hmC enrichment in different data sources, whereas the differences in control groups may be attributed to geographic disparity since all public controls were from Stanford blood center.

Besides, we calculated the 5hmC level of clinically known but nonspecific markers for lung cancer in control and tumor groups, including CEA, CA125 (*MUC16*), NSE (*ENO2*), and CYFRA21-1 (*KRT19*). As shown in Figure S1F, in all genes except *ENO2* (*P* = 8.473E−4), significantly higher 5hmC levels were observed in tumor samples.

**Figure 3:**
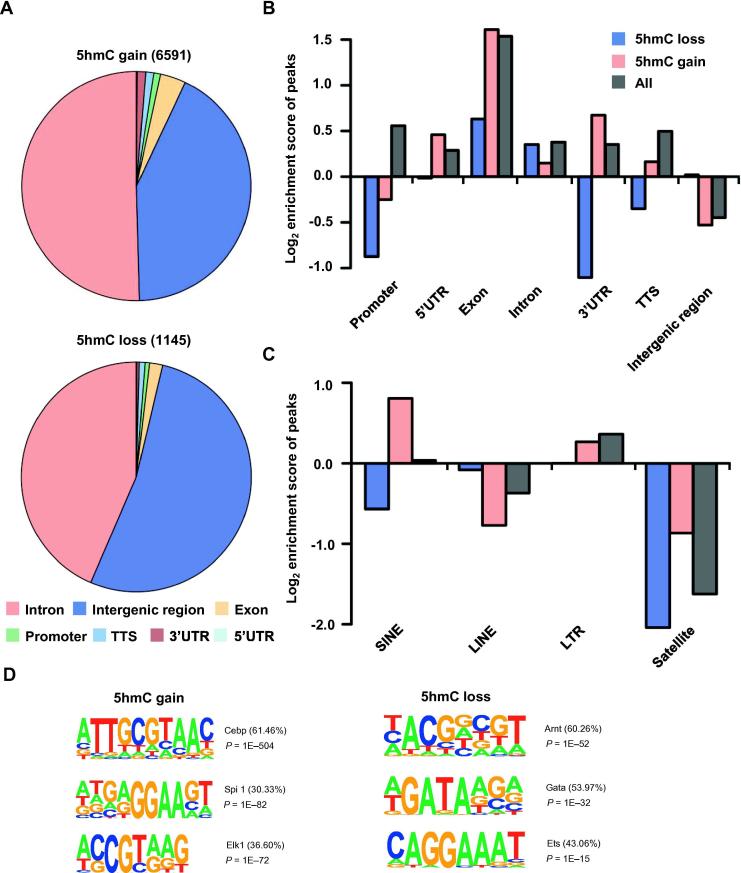
**Genome-wide distribution of DhMRs in blood samples from healthy controls and NSCLC patients** **A.** Distribution of DhMRs in the genomic elements examined. The top panel shows the genomic elements distribution of 5hmC gain regions in tumor samples versus control samples; the bottom panel shows the distribution of 5hmC loss regions. **B.** Normalized enrichment score of DhMRs across distinct genomic regions relative to expected. Enrichment scores of gain or loss regions of 5hmC in tumor samples versus control samples and all 5hmC regions were calculated with positive values indicating enriched more than expected. **C.** Normalized enrichment score of DhMRs in different repeat regions relative to expected. **D.** Top enriched known transcription factor binding motifs detected in DhMRs (left: 5hmC gain; right: 5hmC loss). Motif information was obtained from the Homer motif database. The value in parenthesis represents the percentage of target sequences enriched with the binding motif of the indicated transcription factor. TTS, transcription termination site; DhMR, differentially hydroxymethylated region; Cebp, CCAAT/enhancer binding protein; Spi1, spleen focus forming virus (SFFV) proviral integration oncogene; Gata, GATA binding protein; Elk1, ETS domain-containing protein; Arnt, aryl hydrocarbon receptor nuclear translocator; SINE, short interspersed nuclear element; LINE, long interspersed nuclear element; LTR, long terminal repeat.

To further explore the differences between control and tumor samples, we identified 7736 differentially hydroxymethylated regions (DhMRs) including 6591 5hmC gain regions and 1145 5hmC loss regions in the tumor group compared to the controls. Most of DhMRs (85%) in tumor samples belong to 5hmC gain regions. A large fraction of DhMRs was located in intron regions (50.43%) (Figure 3A). Among all regions examined, the most significant enrichment was found in exons (Figure 3B). Moreover, 5hmC gain regions were particularly enriched in UTRs with log_2_ enrichment score of 0.459/−0.016 in the peaks of 5′UTR (gain/loss), 0.674/−1.105 in the 3′UTR (gain/loss) but lost in intergenic regions (−0.53) compared to 5hmC lost regions (0.02) (Figure 3B). Meanwhile, we found that 5hmC gain regions showed significant enrichment on short interspersed nuclear elements (SINE) compared to all hMRs (Figure 3C). All these results indicated that there is a marked difference in 5hmC profiles of cfDNA between healthy and lung cancer individuals. To better understand the correlation of 5hmC changes with potential interactions of binding proteins, we performed motif enrichment analysis in DhMRs. The 5hmC gain regions were enriched with CCAAT/enhancer binding protein epsilon (Cebp) motifs (*P* = 1E−504), which was highly correlated with transcriptional mis-regulation in cancer pathways [30], [31]. Conversely, the motif of the aryl hydrocarbon receptor nuclear translocator (Arnt), a co-factor that participates in transcriptional regulation by hypoxia-inducible factor 1 and promotes the gene expression during xenobiotic metabolism, was significantly enriched in 5hmC loss regions (*P* = 1E−52) (Figure 3D). Thus, NSCLC patients and healthy controls showed differences in both 5hmC enrichment and potentially interacting binding proteins. Based on the DhMRs of cfDNA, these two groups could be readily separated.

### Gene bodies and promoter regions are hyper-hydroxymethylation in tumor groups

**Figure 4:**
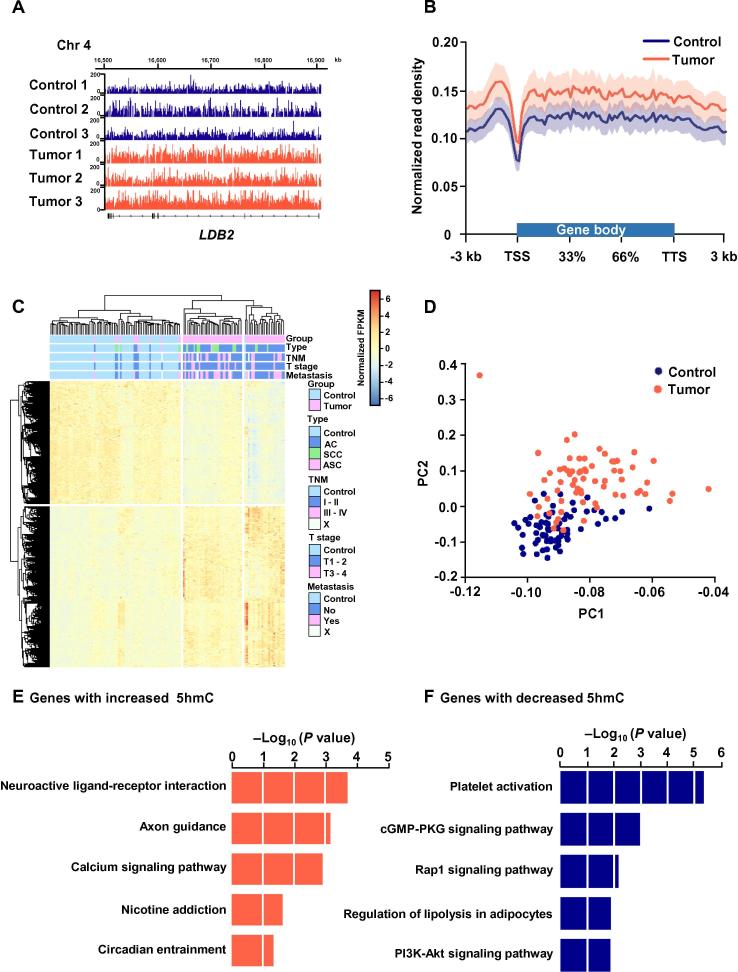
**Identified DhMGs as candidate biomarkers to distinguish blood samples from healthy controls and NSCLC patients** **A.** Genome browser view of the cell-free 5hmC distribution in *LDB2* (one example of differentially-methylated genes) loci in control and tumor samples. The scale represents the rage of normalized read count. **B.** Differentially methylated metagene profiles of cell-free 5hmC in control and tumor samples. Shaded area indicates the upper and lower quartiles. **C.** Heatmap of 2459 DhMGs in control and tumor samples. Hierarchical clustering was performed across genes and samples. **D.** PCA plot of DhMGs FPKM from 67 control and 66 tumor samples. **E.** KEGG enrichment analysis of genes with significant 5hmC increase in tumor samples. **F.** KEGG enrichment analysis of genes with significant 5hmC decrease in tumor samples. LDB2, LIM domain binding 2; AC, adenocarcinoma; SCC, squamous cell carcinoma; ASC, adenosquamous carcinoma; DhMG, hyper-hydroxymethylation among the differential gene. X means data not available for classification of TNM stages or metastasis status.

To further search for the candidate genes with differential 5hmC modification between these two groups, we detected differentially regulated 5hmC genes by DESeq2 package (|FC| > 1.5 and adjusted *P* < 1E−5) and identified 2459 differential 5hmC genes (1396 up-regulated and 1063 down-regulated genes) based on the FPKM of each gene in the tumor group compared to the control group. To illustrate the DhMRs between two groups, we took *LDB2* for example (Figure 4A). The metagene profiles also showed a global hyper-hydroxymethylation among the differential genes (DhMGs) in tumor samples (Figure 4B). Furthermore, unsupervised hierarchical clustering analysis revealed apparent separation between lung cancer and healthy control samples (Figure 4C). Similarly, the unbiased PCA also demonstrated distinct signatures that could separate these two groups (Figure 4D). KEGG functional enrichment analysis showed that up-regulated DhMGs in the lung cancer group are mainly enriched in nicotine addition, calcium signaling pathway, and circadian entrainment pathways, which are closely associated with cancer development [32], [33] (Figure 4E). Genes with decreased 5hmC signal were enriched in several cancer- and metastasis-related pathways including platelet activation pathway, cGMP-PKG signaling pathway, Rap signaling pathway, and PI3K-Akt signaling pathway [34], [35] (Figure 4F).

Besides DhMGs, aberrant 5hmC enrichment in promoter proximal regions could also be relevant to the carcinogenic process [36], [37]. After calculating the normalized read density around transcription start site (TSS), we found that the average profile of the 5hmC level showed obvious 5hmC gain in the tumor group (Figure S2A). By comparing the differentially hydroxymethylated promoters (DhMPs) between tumor and control samples using the same approach in DhMG identification, we identified 1344 DhMPs, including 857 5hmC gain and 487 5hmC loss genes. Similar to the gene bodies, higher abundance of 5hmC was also observed in gene promoter regions, such as *FBXL7* (Figure S2B). The hierarchical clustering analysis and unbiased PCA indicated that DhMPs could also separate these two groups well (Figure S2C and S2D). Interestingly, KEGG functional enrichment analysis for genes with DhMPs revealed different functions related to cancer development from that of DhMGs (Figure S2E and S2F) [38], [39], which may result from the differential mechanisms of gene expression regulation during cancer development [20]. In light of the results above, we infer that DhMGs and DhMPs of cfDNA could be highly associated with carcinogenic process and may serve as potential candidates for further biomarker validation.

### Six aberrant hydroxymethylated genes are highly conserved in controls compared with tumor samples

**Figure 5:**
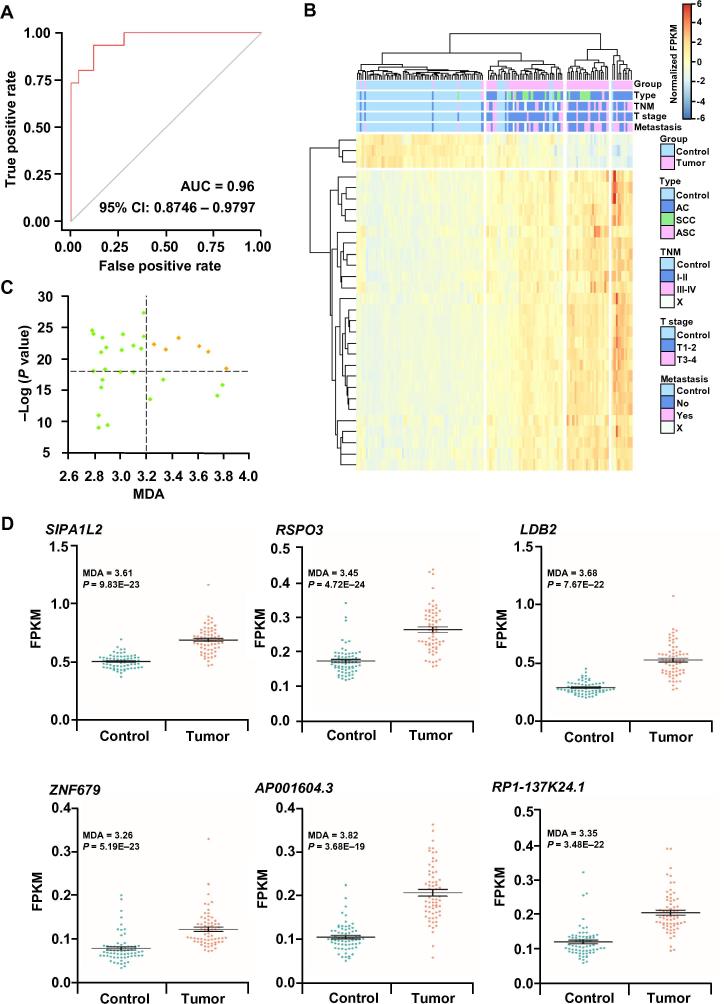
**Performance of potential hydroxymethylation markers for lung cancer** **A.** ROC of the diagnostic prediction model with potential hydroxymethylation markers in the validation dataset (24 control and 17 tumor samples). The red and gray lines represent validation and standard accuracy curves, respectively. **B.** Unsupervised hierarchical clustering of the top 30 potential hydroxymethylation markers in tumor and control groups. **C.** Scatterplot showing the MDA and the significance of two-tailed *t-*tests for the top 30 potential markers. Yellow dots refer to significant differential genes. **D.** The hydroxymethylation level (FPKM) of the six potential genes in the control and tumor groups. AUC, area under curve; CI, confidence interval; AC, adenocarcinoma; SCC, squamous cell carcinoma; ASC, adenosquamous carcinoma; MDA, mean decrease accuracy; SIPA1L2, signal induced proliferation associated 1 like 2; RSPO3, R-spondin 3; LDB2, LIM domain binding 2; ZNF679, zinc finger protein 679. X means data not available for classification of TNM stages or metastasis status.

Considering the distinct 5hmC signals in DhMGs, we then performed the Random-Forest analysis as a machine classifier to differentiate tumor and control groups based on the detected DhMGs. With the increase in tree numbers the model built, error rates decreased accordingly and tended to be stable at ∼700 (Figure S3A). Using the optimum parameters with 700 trees (see details in the methods), we built the model that was able to differentiate lung cancer patients from healthy controls in the training (AUC = 0.9272, CI: 0.8746–0.9797) and validation dataset (AUC = 0.9600, CI: 0.8582–0.9723) (Figure S3B and Figure 5A). Hierarchical clustering analysis using the top 30 mean decrease accuracy (MDA) differentially modified 5hmC genes could well separate lung cancer patients from healthy controls (Figure 5B).

To further select the most reliable hydroxymethylation marker genes, we used both MDA and the significance (*P* value) of two-tailed t-tests to filter 30 candidate genes (Figure SC3, S3D and 5C). The top six potential genes were *SIPA1L2* (MDA = 3.61, *P* = 9.82615E−23), *RSPO3* (MDA = 3.45, *P* = 4.72349E−24), *LDB2* (MDA = 3.68, *P* = 7.66646E−22), *ZNF679* (MDA = 3.26, *P* = 5.1857E−23), *AP001604.3* (MDA = 3.82, *P* = 3.68029E−19), and *RP1-137K24.1* (MDA = 3.35, *P* = 3.48252E−22) (Figure 5D). All these six selected markers had obvious differences in 5hmC enrichment in most of the cancer patients compared to the normal controls. These results suggest that the aberrant hydroxymethylation levels of these six genes could be the potential diagnostic biomarkers for lung cancer.

**Figure 6:**
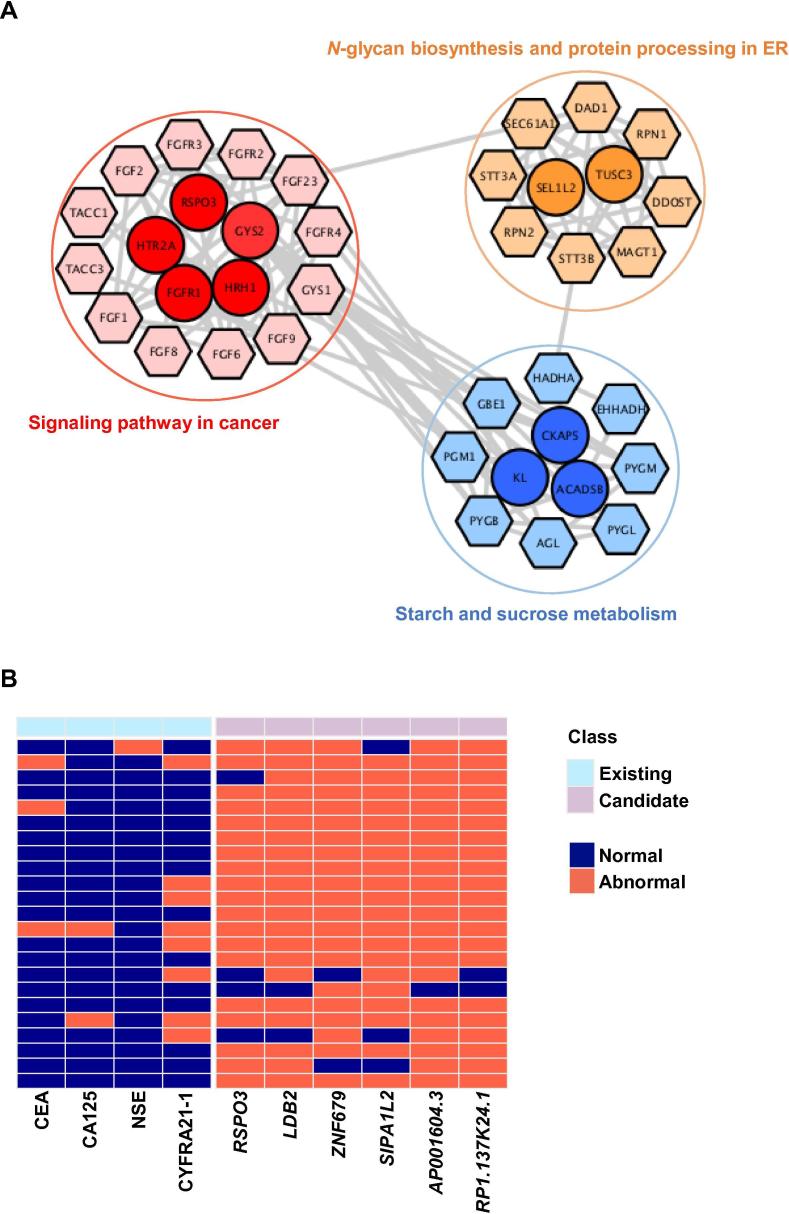
**Candidate biomarkers closely associated with cancer development with high accuracy** **A.** The PPI network analysis and functional enrichment analysis with the potential target proteins of the candidate biomarkers. Top functions were selected to be presented. Network nodes represent the proteins, lines represent the protein interaction. Circle nodes in deep colors indicate candidate biomarkers, whereas hexagon nodes in light colors indicate the potential target proteins of candidate biomarkers. **B.** The performance of existing and candidate biomarkers for evaluating NSCLC patients. Each row represents a NSCLC patient, dark blue and tomato boxes represent a normal and abnormal range of biomarkers (normal range of existing biomarkers levels in serum: CEA < 5 μg/l, CA125 < 35 U/ml, NSE < 17.00 ng/ml, CYFRA21_1 0.1–3.3 ng/ml). CEA; carcinoembryonic antigen; CA125, carbohydrate antigen 125; NSE, neuron-specific enolase; CYFRA21-1, cytokeratin 19 fragment.

Next, we sought to investigate whether the candidate marker genes are associated with carcinogenesis. We performed protein–protein interaction (PPI) and functional enrichment analyses using the top 100 candidate genes from our classifier (Table S2). We found that the selected candidates were mainly enriched in the signaling pathways related to cancer, including Rap1, MAPK and PI3K-Akt signaling pathways, as well as the metabolic pathways, such as starch and sucrose metabolism, *N*-glycan biosynthesis, and protein processing in the endoplasmic reticulum (ER) (Figure 6A).

To further verify the sensitivity of the 5hmC biomarkers selected, we compared the 5hmC candidate biomarkers filtered in the current study and the known clinical biomarkers. We found that 43.48% (10/23) individuals showed negative results using any of the traditional markers (misjudgment), whereas exhibited positive results using all of the six candidate genes selected (well-judged). Meanwhile, 86.96% (20/23) individuals showed at least 2 positive results based on the test of six candidate genes (Figure 6B and Table S3). These results suggest that cfDNA 5hmC of these six candidate genes are more sensitive for predicting lung cancer than the traditional ones and may potentially serve as sensitive and specific diagnostic biomarkers for NSCLC.

## Discussion

Recent studies demonstrate that 5hmC, a relative stable intermediate product of active DNA demethylation, plays a critical role in gene expression regulation and is also regarded as a novel epigenetic biomarker for cancer diagnosis and prognosis [20], [21], [22]. In this study, we utilized a sensitive 5hmC-Seal method [27] to generate the genome-wide profiles of cell-free 5hmC in NSCLC patients and healthy controls. We have identified the robust NSCLC-associated 5hmC signatures with significant 5hmC gain in gene bodies and promoter regions in NSCLC patients. Moreover, we also find that genes with 5hmC gain are highly associated with cancer occurrence and progression. Meanwhile, we further discover potential 5hmC-based biomarkers in circulating cfDNA of NSCLC via machine-learning-based tumor classifiers. Overall, our findings illustrate that 5hmC signatures of cfDNA have the potential to serve as biomarkers for NSCLC, the performance of which could be largely improved by recruiting more patients in the future studies.

Alterations in the cancer-associated 5hmC signature change in plasma cfDNA are highly predictive for several types of human cancers [24], [25], [26]. By sequencing the genome-wide 5hmC in the cfDNA from 49 cancer patients, including 15 lung cancer, 10 hepatocellular carcinoma, 7 pancreatic cancer, 4 glioblastoma, 5 gastric cancer, 4 colorectal cancer and 4 breast cancer patients, Song et al. have reported a progressive global loss of cell-free 5hmC in lung cancer [25]. However, hepatocellular carcinoma and pancreatic cancer show both enriched and depleted 5hmC genes compared to healthy controls. Li et al. have detected the genome-wide distribution of 5hmC in cfDNA from 90 healthy individuals and 260 patients with colorectal, gastric, liver, pancreatic, or thyroid cancer [26]. They further identify 5hmC-based biomarkers derived from circulating cfDNA with high sensitivity and specificity for colorectal and gastric cancers. Our previous study has revealed the esophageal cancer-associated 5hmC changes in plasma cfDNA, and discovered that 5hmC biomarkers could be used for early detection of esophageal cancer [24]. Taken together, these findings indicate that cell-free 5hmC sequencing may provide a promising noninvasive approach for cancer diagnosis and prognosis.

Our findings that global cell-free 5hmC gains in both gene body and promoter regions in NSCLC patients compared to healthy controls (Figures 2D, 4B, 4C, S2A, and S2C) are inconsistent with the previous study reported by Song and his colleagues [25]. This may be due to the geographic disparity for the normal controls. As for public data [25], the 15 lung cancer patients were recruited in a West China Hospital but 8 healthy controls (1 corresponding input) were obtained from Stanford blood center. It has been shown that ethnic differences in both genomic and epigenetic polymorphisms exist, which presumably contribute to the markedly distinctive features of cancer profiles in different populations, resulting in varied modalities for diagnosis, prognosis, and treatment guidance [40], [41], [42]. Thus, whether different ethnic groups display distinct cfDNA 5hmC features and thereby lead to distinguished approaches for cancer diagnosis in clinical setting remain unclear and need further examination.

Previous studies indicate that aberrant 5hmC enrichment in the promoter regions is also associated with the carcinogenic process [36], [37]. Uribe-Lewis et al. reported that 5hmC in promoter regions could be used as colon cancer markers [36]. Additionally, DhMPs identified from their studies could well separate cancer patients and controls. Moreover, distinct differences in DhMPs of cfDNA between tumor and control groups were also demonstrated in our study (Figure S2A–D). In addition, the gene sets affected by the differential 5hmC modification in the promoters were strongly associated with cancer development (Figure S2E and S2F). Further studies should be performed to evaluate the potential value and accuracy of DhMPs in tumor classification and detection using large-scale tumor samples with multiple histological tissue types.

Considering the highly heterogeneous nature of lung cancer, large-scale clinical studies are required to identify disease-specific cell-free 5hmC signatures and further validate their sensitivity, specificity, and accuracy in the early diagnosis of lung cancer. It has been reported that about 25% of patients with stage I lung cancer will have recurrent disease due to occult metastasis [9]. Thus, 5hmC-based biomarkers may also have the potential value to classify early-stage (IA and IB) lung cancers into subtypes with low risk and high risk recurrence pending with appropriate treatment, such that the post-surgery adjuvant therapy should mandatorily be given to the patients with higher risk of metastasis. Collectively, the detection of 5hmC-based biomarkers in the cfDNA may offer a non-invasive and easily accessible method for early diagnosis and treatment of human cancers, and also potentially for other diseases such as neurodegenerative, cardiovascular, and metabolic diseases.

## Conclusion

In this study, we have generated the 5hmC profiles of cfDNA from Chinese NSCLC patients and detected the large-scale 5hmC gains in both gene bodies and promoter regions in the tumor group compared with healthy controls. Six potential biomarkers are further identified to be highly conserved in controls compared with heterogeneous tumor samples, and moreover, have a higher sensitivity in disease diagnosis than classical biomarkers. Our findings are potentially valuable in the development of new strategies for diagnosis and therapeutic treatment of NSCLC.

## Materials and methods

### Patient characteristics

In total, 74 patients with NSCLC were enrolled from the First Affiliated Hospital of Zhengzhou University, Zhengzhou, China, from September 2016 to July 2017. Peripheral blood samples from NSCLC patients were obtained preoperatively from the Department of Thoracic Surgery. We excluded patients that received surgery, chemoradiotherapy or immunotherapy within the past six months when sample were collected. After a strict pathological diagnosis and exclusion process, 66 patients with NSCLC were included and subjected to 5hmC sequencing. Cancer stages were classified according to the Eighth Edition Lung Cancer Stage Classification in AJCC/UICC cancer staging manuals [43]. The information of classification of TNM stages and lymphatic metastasis is not available for 4 NSCLC patients. A total of 67 healthy control samples were retrieved from the study by Tian and his colleagues [24], which were also collected from the First Affiliated Hospital of Zhengzhou University between September 2016 and July 2017. To minimize the age and gender impacts, we have selected the controls that are comparable with tumor group. This study was approved by the Institutional Review Board of the First Affiliated Hospital of Zhengzhou University. All subjects provided written informed consent according to the institutional guidelines.

### Blood sample processing

Plasma samples were obtained from peripheral blood (about 4 ml per sample) by taking the supernatant after centrifugation twice at 1350*g* for 12 min and once at 13,500*g* for 12 min at 4 °C. The QIAamp Circulating Nucleic Acid Kit (55114, Qiagen, Valencia, CA, USA) was used for cfDNA extraction by following the manufacturer’s manual.

### 5hmC library construction and sequencing

5hmC library construction was performed as described previously [24]. Briefly, the cfDNA was ligated with sequencing compatible adaptors. Next, ligated DNA was incubated in a 25-µl reaction solution containing HEPES buffer (50 mM, pH 8.0), MgCl_2_ (25 mM), N_3_-UDP-Glc (100 µM, Active Motif, Carlsbad, CA, USA), and β-glucosyltransferase (1 µM, Thermo, Waltham, MA, USA) for 1 h at 37 °C. Then, 1 µl DBCO-PEG4-DBCO (4.5 mM, Click Chemistry Tools, Scottsdale, AZ, USA) was added and incubated for 2 h at 37 °C. Subsequently, the Micro Bio-Spin 30 Column (Bio-Rad, Richmond, CA, USA) was used to purify the DNA. Thereafter, C1 streptavidin beads (5 µl, Life Technologies, Gaithersburg, MD, USA) were added. After incubation with DNA for 15 min at room temperature, the beads underwent eight 5-min washes. The 5hmC-containing cfDNA fragments were amplified with 14–16 cycles of PCR amplification (initial denaturing at 98 °C for 45 s, followed by 14–16 cycles of denaturing at 98 °C for 15 s, annealing at 60 °C for 30 s, extension at 72 °C for 30 s, and a final extension at 72 °C for 60 s). The amplified product was purified using AMPure XP beads and used as the library for high-throughput sequencing analysis on the Illumina NextSeq 500 platform.

The 5hmC-seq data were deposited in the Genome Sequence Archive [44] in BIG Data Center [45], Beijing Institute of Genomics (BIG), Chinese Academy of Sciences, under accession number PRJCA000816 that are publicly accessible at http://bigd.big.ac.cn/gsa.

### Mapping and differentially modified regions detection

For the sequencing data, Trimmomatic (version 0.33) [46] was used to trim off adaptor sequences, and reads <35 nt in length were filtered out. The remaining reads were mapped to the human genome (version hg19) using Bowtie 2 (version 2.2.9) [47]. The mapped reads with quality score ≥20 were retained for the subsequent analysis.

Identification of 5hmC-enriched regions (hMRs) was performed using MACS2 (version 2.1.1) [48]. Genomic annotations of hMRs were performed using the “intersect” function of BEDTools (version 2.26.0) [49] and the genome-wide distribution of 5hmC was visualized using Circos [50]. The metagene profile was generated using ngsplot [51]. Peaks with high enrichment and significance (*q* < 1E−12; fold enrichment >8) in all samples were considered as highly reliable peaks and were combined into one unified catalogue by the ‘‘mergePeak’’ function from HOMER (version 4.9.1) (merged peaks: 266,514) [52]. BEDTools [49] was then used to calculate the tag counts of merged highly reliable peaks in all samples.

### Detection of differential genes and functional analysis

Paired-end reads were converted into BedGraph format normalized by BEDTools and visualized using the Integrated Genomics Viewer [53], [54]. 5hmC FPKM were calculated using the fragment counts in each of the Ensembl gene bodies (parameters used: –F 0.3 –c) and promoter regions (defined as 1 kb upstream of TSS for each Ensembl gene) obtained by BEDTools. After filtering out genes in chromosomes X and Y, differentially modified genes in the autosomes between samples from patients with NSCLC and healthy controls were identified using DESeq2 package (|FC| > 1.5 and adjusted *P* < 1E−5). *De novo* motif analysis around DhMRs was performed using HOMER (version 4.4) [52]. Furthermore, significant differential genes were analyzed using the principle component analysis (PCA). Hierarchical clustering and heatmap analyses were performed using the R Statistical Package (version 3.4.1). KEGG pathway analysis was performed using DAVID Bioinformatics Resources 6.8 [55], [56]. PCA and hierarchical clustering analyses for DhMPs were also performed in R. The PPI network and functional enrichment annotation were generated with the top 100 genes identified with the classifier using the Search Tool for the Retrieval of Interacting Genes (STRING) database [57].

### Classifier construction

The Random-Forest model [58] was applied as a machine classifier to calculate the mean decrease accuracy (MDA) of differential genes in tumor and control groups, using the Random Forest library in the R Statistical Package. We set up different combinations of parameters for the number of trees and the genes that the algorithm selected every time to find the optimum combination (ntree = 700, mtry = 48). The training and validation datasets of all differential genes were selected randomly with the proportion of 7:3. To ensure the significance of each potential marker, we used two-tailed *t*-tests to obtain the *P* value for the top 30 genes, and defined genes with an MDA >3.2 and −log_10_ (*P* value) >18 as significantly different. Prism (GraphPad, La Jolla, CA) was used to visualize the different 5hmC levels between tumor and control groups.

## Authors’ contributions

YY and XT conceived this project. XT, BS, and XL designed and supervised the experiments. XL, JZ, YX, ZQ, RL, YW, and XZ enrolled patients, collected blood samples, and analyzed clinical data. BS, CG, and XH performed bioinformatics analysis. XH, JZ, CG, XT, and BS wrote the manuscript with input from all authors. All authors read and approved the final manuscript.

## Competing interests

The authors have declared that they have no competing interests.

## Supplementary material

Supplementary Figure S1 **Overview of 5hmC level in blood samples of different data sources**. **A.** Genome-wide (except chromosome X and Y) distribution of 5hmC in control and tumor samples. Chromosomes are shown in different colors and 5hmC enrichment levels are shown in lines. Significant peaks (q < 1E−10) are shown in the Circos plot for each sample. **B.** The mean number of 5hmC peaks located across distinct genomic regions in healthy and NSCLC groups (promoter is defined as 1 kb upstream of TSS). The number of hMRs in intergenic regions were omitted due to its tiny number. **C.** Metagene profiles of cell-free 5hmC in blood samples from healthy controls and patients with NSCLC, along with online data of control, tumor, and unenriched input cfDNA samples from Song et al [25] (GSE81314). Shaded area indicates the upper and lower quartile. **D.** Correlation between 5hmC FPKM with 5hmC level measured within each gene, and correlation coefficients are shown and colored from blue to red to indicate low to high correlation. **E.** PCA plot of 5hmC FPKM from control and tumor samples in our own data and public data of NSCLC. **F.** Boxplot shows 5hmC level of clinically known tumor markers in our control and tumor samples. TSS, transcription start site; TTS, transcription termination site; hMR, 5hmC-enriched region; CEA; carcinoembryonic antigen; MUC16 (CA125), mucin 16, cell surface associated; ENO2 (NSE), enolase 2; KRT19 (CYFRA21-1), keratin 19.

Supplementary Figure S2 **5hmC in promoter regions was abnormal in tumor group**. **A.** Average metagene profiles plot near TSS of cell-free 5hmC in control and tumor samples. Shaded area indicates the upper and lower quartiles. **B.** Genome browser view of the cell-free 5hmC distribution in FBXL7 promoter loci in control and tumor samples. The differentially methylated promoter region was marked with a green box. The scale represents the rage of normalized read count. **C.** Heatmap of 1344 DhMPs in control and tumor samples. **D.** PCA plot of DhMPs 5hmC FPKM from 67 control and 66 tumor samples. **E.−F.** KEGG enrichment analysis of promoters with signiﬁcant 5hmC increase (E) or decrease (F) in tumor samples. TSS, transcription start site; FBXL7, F-box and leucine-rich repeat protein 7; AC, adenocarcinoma; SCC, squamous cell carcinoma; ASC, adenosquamous carcinoma; DhMP, differentially hydroxymethylated promoter. X means data not available for classification of TNM stages or metastasis status.

Supplementary Figure S3 **Screening process of 5hmC markers using Random-Forest analysis**. **A.** The tendency of error rates in tumor and control groups by different trees Random-Forest built. **B.** Summary tables of the machine-learning classifier in the training dataset. The red boxes represents well-judged number. **C.** Composite diagram showing MDA for the top 30 potential markers. **D.** Composite diagram showing the significance of the *t*-test for the top 30 potential markers. OOB, out of bag error; MDA, mean decrease accuracy.
