# Supplementary figures and images for: 5-Hydroxymethylome in Circulating Cell-free DNA as A Potential Biomarker for Non-small-cell Lung Cancer
Source: Genomics Proteomics Bioinformatics. 2018 Jul 18;16(3):187–99. doi: 10.1016/j.gpb.2018.06.002 (PMC6076378; doi:10.1016/j.gpb.2018.06.002)

**A**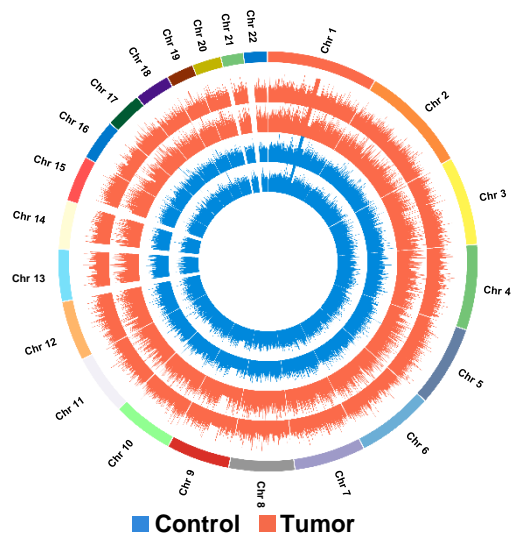**B**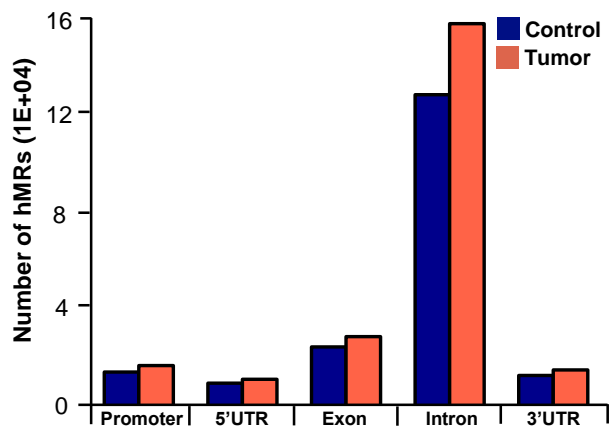**C**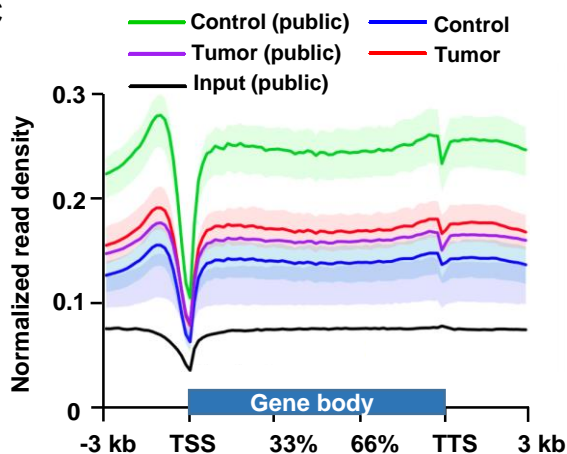**D**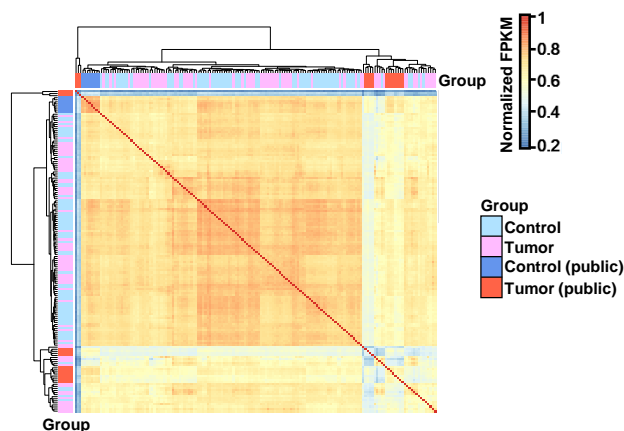**E**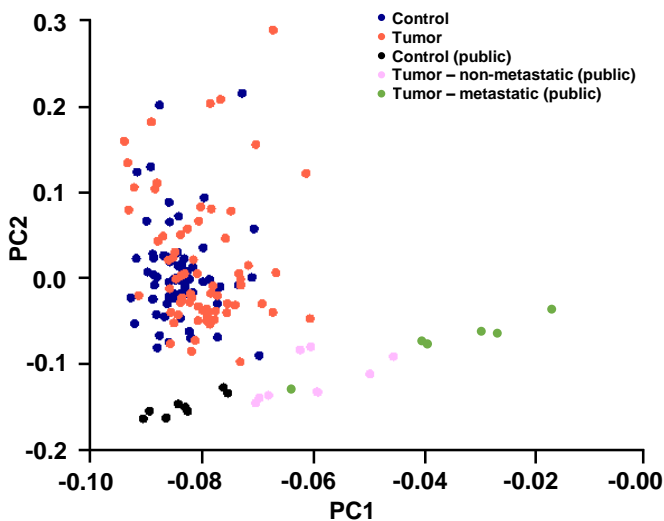**F**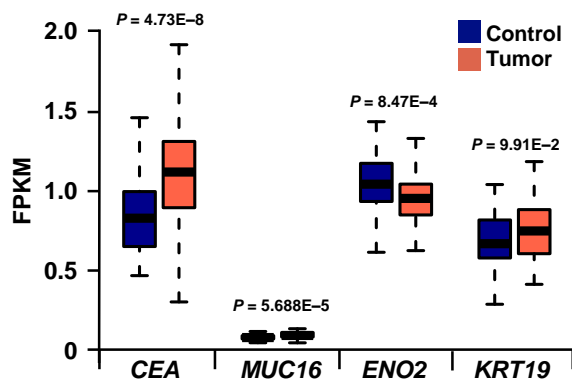

Supplement: Supplementary Figure S1 — Overview of 5hmC level in blood samples of different data sources. A. Genome-wide (except chromosome X and Y) distribution of 5hmC in control and tumor samples. Chromosomes are shown in different colors and 5hmC enrichment levels are shown in lines. Significant peaks (q < 1E−10) are shown in the Circos plot for each sample. B. The mean number of 5hmC peaks located across distinct genomic regions in healthy and NSCLC groups (promoter is defined as 1 kb upstream of TSS). The number of hMRs in intergenic regions were omitted due to its tiny number. C. Metagene profiles of cell-free 5hmC in blood samples from healthy controls and patients with NSCLC, along with online data of control, tumor, and unenriched input cfDNA samples from Song et al [25] (GSE81314). Shaded area indicates the upper and lower quartile. D. Correlation between 5hmC FPKM with 5hmC level measured within each gene, and correlation coefficients are shown and colored from blue to red to indicate low to high correlation. E. PCA plot of 5hmC FPKM from control and tumor samples in our own data and public data of NSCLC. F. Boxplot shows 5hmC level of clinically known tumor markers in our control and tumor samples. TSS, transcription start site; TTS, transcription termination site; hMR, 5hmC-enriched region; CEA; carcinoembryonic antigen; MUC16 (CA125), mucin 16, cell surface associated; ENO2 (NSE), enolase 2; KRT19 (CYFRA21-1), keratin 19. [file mmc1.pdf]

**A**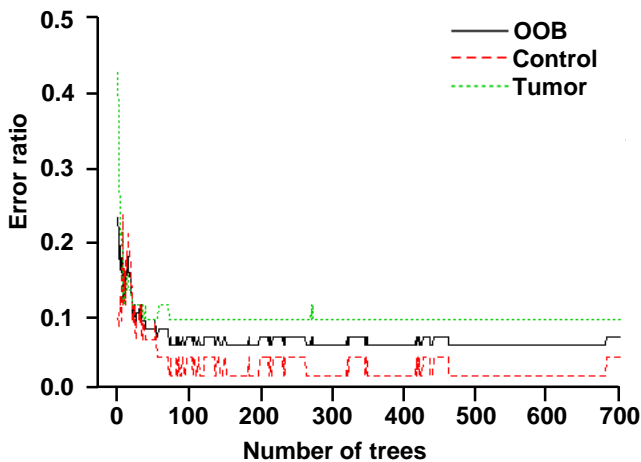**B**

| Type    | Control | Tumor | Class error |
|---------|---------|-------|-------------|
| Control | 40      | 2     | 0.0476      |
| Tumor   | 5       | 46    | 0.0980      |

**C**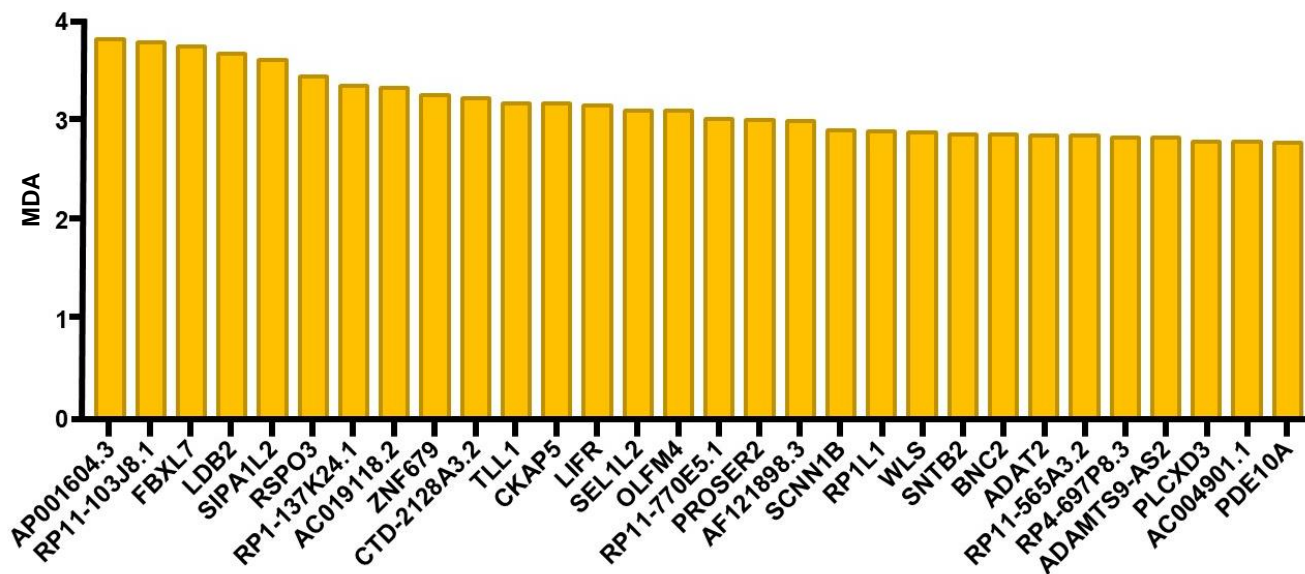**D**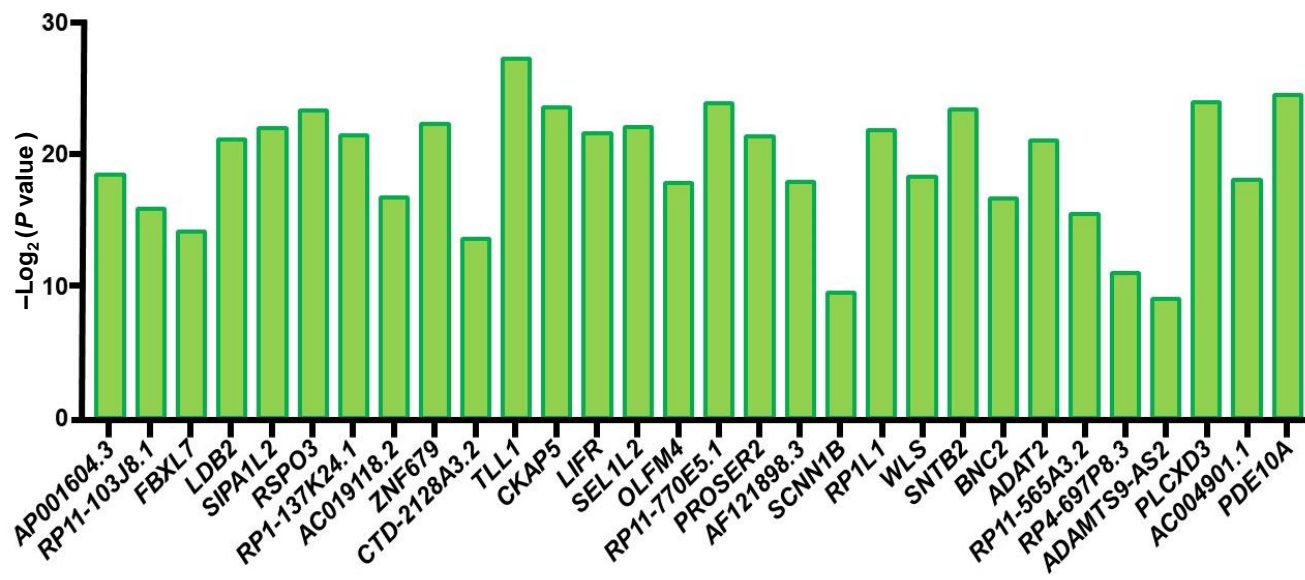

Supplement: Supplementary Figure S3 — Screening process of 5hmC markers using Random-Forest analysis. A. The tendency of error rates in tumor and control groups by different trees Random-Forest built. B. Summary tables of the machine-learning classifier in the training dataset. The red boxes represents well-judged number. C. Composite diagram showing MDA for the top 30 potential markers. D. Composite diagram showing the significance of the t-test for the top 30 potential markers. OOB, out of bag error; MDA, mean decrease accuracy. [file mmc3.pdf]
