# Supplementary material for: 5-Hydroxymethylome in Circulating Cell-free DNA as A Potential Biomarker for Non-small-cell Lung Cancer
Source: Genomics Proteomics Bioinformatics. 2018 Jul 18;16(3):187–99. doi: 10.1016/j.gpb.2018.06.002 (PMC6076378; doi:10.1016/j.gpb.2018.06.002)
Supplement: Supplementary Figure S2 — 5hmC in promoter regions was abnormal in tumor group. A. Average metagene profiles plot near TSS of cell-free 5hmC in control and tumor samples. Shaded area indicates the upper and lower quartiles. B. Genome browser view of the cell-free 5hmC distribution in FBXL7 promoter loci in control and tumor samples. The differentially methylated promoter region was marked with a green box. The scale represents the rage of normalized read count. C. Heatmap of 1344 DhMPs in control and tumor samples. D. PCA plot of DhMPs 5hmC FPKM from 67 control and 66 tumor samples. E.−F. KEGG enrichment analysis of promoters with signiﬁcant 5hmC increase (E) or decrease (F) in tumor samples. TSS, transcription start site; FBXL7, F-box and leucine-rich repeat protein 7; AC, adenocarcinoma; SCC, squamous cell carcinoma; ASC, adenosquamous carcinoma; DhMP, differentially hydroxymethylated promoter. X means data not available for classification of TNM stages or metastasis status. [file mmc2.pdf]

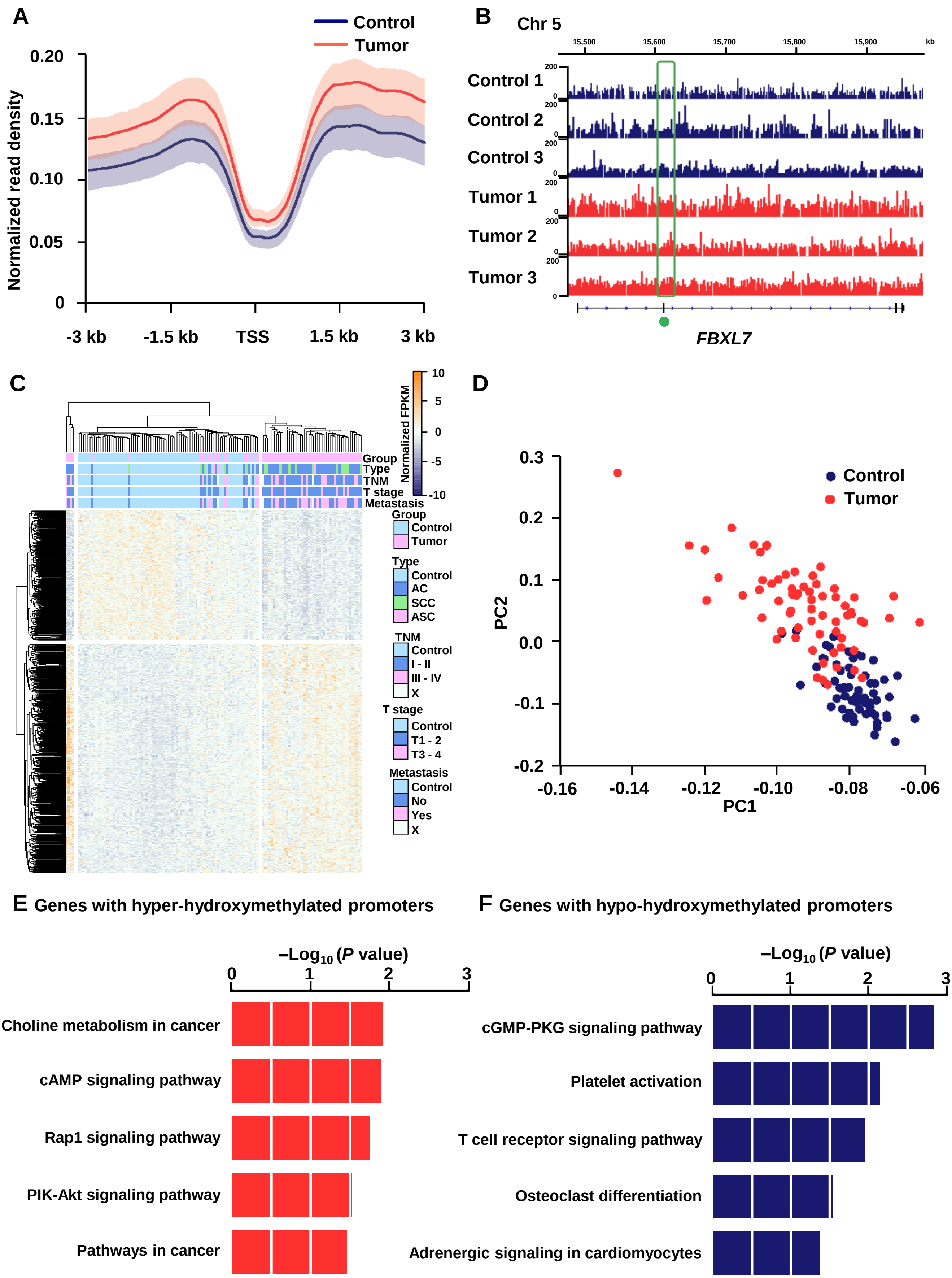

A
Control
Tumor
Normalized read density
0.20
0.15
0.10
0.05
0
-3 kb
-1.5 kb
TSS
1.5 kb
3 kb
B
Chr 5
15,500
15,600
15,700
15,800
15,900
kb
Control 1
Control 2
Control 3
Tumor 1
Tumor 2
Tumor 3
FBXL7
C
Normalized FPKM
10
5
0
-5
-10
Group
Type
TNM
T stage
Metastasis
Group
Control
Tumor
Type
Control
AC
SCC
ASC
TNM
Control
I - II
III - IV
X
T stage
Control
T1 - 2
T3 - 4
Metastasis
Control
No
Yes
X
D
Control
Tumor
PC2
PC1
0.3
0.2
0.1
0.0
-0.1
-0.2
-0.16
-0.14
-0.12
-0.10
-0.08
-0.06
E Genes with hyper-hydroxymethylated promoters
–Log10 (P value)
0
1
2
3
Choline metabolism in cancer
cAMP signaling pathway
Rap1 signaling pathway
PIK-Akt signaling pathway
Pathways in cancer
F Genes with hypo-hydroxymethylated promoters
–Log10 (P value)
0
1
2
3
cGMP-PKG signaling pathway
Platelet activation
T cell receptor signaling pathway
Osteoclast differentiation
Adrenergic signaling in cardiomyocytes
