# Supplementary material for: 5-Hydroxymethylome in Circulating Cell-free DNA as A Potential Biomarker for Non-small-cell Lung Cancer
Source: Genomics Proteomics Bioinformatics. 2018 Jul 18;16(3):187–99. doi: 10.1016/j.gpb.2018.06.002 (PMC6076378; doi:10.1016/j.gpb.2018.06.002)
Supplement: Supplementary Table S1 [file mmc4.docx]

**Table S1 Clinicopathological characteristics of NSCLC patients and healthy individuals**

|  | **Healthy controls (n = 67)** | **NSCLC patients (n = 66)** |
| --- | --- | --- |
| **Age (year)** |  |  |
| Mean ± SD | 55 ± 7.9 | 59 ± 9.0 |
| Median (range) | 54 (30－72) | 60.5 (31－76) |
| **Gender** |  |  |
| Male | 39 | 35 |
| Female | 28 | 31 |
| **Cancer type** |  |  |
| Adenocarcinoma | - | 46 |
| Squamous carcinoma | - | 17 |
| Adenosquamous carcinoma | - | 3 |
| **TNM stage** |  |  |
| Ⅰ | - | 26 |
| Ⅱ | - | 17 |
| Ⅲ | - | 18 |
| IV | - | 1 |
| Data not available | - | 4 |
| **T stage** |  |  |
| 1 | - | 21 |
| 2 | - | 33 |
| 3 | - | 7 |
| 4 | - | 5 |
| **Lymphatic metastasis** |  |  |
| Metastatic | - | 28 |
| Non-metastatic | - | 34 |
| Data not available | - | 4 |
| **CEA** |  |  |
| Positive | 0 | 9 |
| Negative | 54 | 31 |
| Data not available | 13 | 26 |
| **CA125** |  |  |
| Positive | 0 | 2 |
| Negative | 42 | 32 |
| Data not available | 25 | 32 |
| **NSE** |  |  |
| Positive | 0 | 2 |
| Negative | 28 | 34 |
| Data not available | 39 | 30 |
| **CYFRA21-1** |  |  |
| Positive | 3 | 11 |
| Negative | 25 | 18 |
| Data not available | 39 | 37 |
| **AFP** |  |  |
| Positive | 0 | 0 |
| Negative | 30 | 48 |
| Data not available | 36 | 19 |
| **CA19-9** |  |  |
| Positive | 0 | 0 |
| Negative | 31 | 29 |
| Data not available | 35 | 38 |
| **CA15-3** |  |  |
| Positive | 0 | 0 |
| Negative | 18 | 32 |
| Data not available | 48 | 35 |

*Note:* CEA, carcinoembryonic antigen; NSE, neuron-specific enolase; CYFRA21-1, cytokeratin 19 fragment; AFP, alpha-fetoprotein; CA125, carbohydrate antigen 125; CA19-9, carbohydrate antigen 19-9; CA15-3, carbohydrate antigen 15-3.
