# Supplementary material for: 5-Hydroxymethylome in Circulating Cell-free DNA as A Potential Biomarker for Non-small-cell Lung Cancer
Source: Genomics Proteomics Bioinformatics. 2018 Jul 18;16(3):187–99. doi: 10.1016/j.gpb.2018.06.002 (PMC6076378; doi:10.1016/j.gpb.2018.06.002)
Supplement: Supplementary Table S2 [file mmc5.docx]

**Table S2 The top 100 important genes obtained from machine learning classifier**

| **Ensembl_ID** | **Locus_tag** | **FPKM (control)** | **FPKM (tumor)** | **MDA** | **MDG** |
| --- | --- | --- | --- | --- | --- |
| ENSG00000231236 | *AP001604.3* | 3.99 | 1.64 | 3.82 | 0.47 |
| ENSG00000270792 | *RP11-103J8.1* | 3.67 | 2.7 | 3.79 | 0.34 |
| ENSG00000183580 | *FBXL7* | 3.68 | 3.11 | 3.75 | 0.46 |
| ENSG00000169744 | *LDB2* | 3.83 | 2.58 | 3.68 | 0.37 |
| ENSG00000116991 | *SIPA1L2* | 3.37 | 2.81 | 3.61 | 0.32 |
| ENSG00000146374 | *RSPO3* | 2.95 | 2.68 | 3.45 | 0.32 |
| ENSG00000248486 | *RP1-137K24.1* | 2.99 | 2.66 | 3.35 | 0.33 |
| ENSG00000234423 | *AC019118.2* | 3.06 | 2.22 | 3.33 | 0.28 |
| ENSG00000197123 | *ZNF679* | 3.12 | 2.3 | 3.26 | 0.15 |
| ENSG00000258902 | *CTD-2128A3.2* | 2.92 | 2.79 | 3.23 | 0.26 |
| ENSG00000038295 | *TLL1* | 3.2 | 2.43 | 3.18 | 0.35 |
| ENSG00000175216 | *CKAP5* | 3.22 | 1.16 | 3.18 | 0.32 |
| ENSG00000113594 | *LIFR* | 2.48 | 2.52 | 3.16 | 0.17 |
| ENSG00000101251 | *SEL1L2* | 3.08 | -0.11 | 3.1 | 0.29 |
| ENSG00000102837 | *OLFM4* | 3.02 | 2.69 | 3.1 | 0.19 |
| ENSG00000253608 | *RP11-770E5.1* | 2.92 | 2.96 | 3.02 | 0.27 |
| ENSG00000148426 | *PROSER2* | 2.94 | 0.77 | 3.01 | 0.28 |
| ENSG00000253500 | *AF121898.3* | 2.46 | 2.52 | 3 | 0.25 |
| ENSG00000168447 | *SCNN1B* | 2.94 | 1.65 | 2.9 | 0.25 |
| ENSG00000183638 | *RP1L1* | 3.02 | 2.08 | 2.89 | 0.26 |
| ENSG00000116729 | *WLS* | 2.7 | 2 | 2.88 | 0.18 |
| ENSG00000168807 | *SNTB2* | 2.46 | 2.19 | 2.86 | 0.25 |
| ENSG00000173068 | *BNC2* | 2.87 | 2.4 | 2.86 | 0.26 |
| ENSG00000189007 | *ADAT2* | 2.4 | 2.69 | 2.85 | 0.15 |
| ENSG00000250590 | *RP11-565A3.2* | 2.63 | 2.61 | 2.85 | 0.21 |
| ENSG00000232900 | *RP4-697P8.3* | 2.72 | 2.64 | 2.83 | 0.24 |
| ENSG00000241684 | *ADAMTS9-AS2* | 2.78 | 2.23 | 2.83 | 0.25 |
| ENSG00000182836 | *PLCXD3* | 2.76 | 2.4 | 2.79 | 0.28 |
| ENSG00000232072 | *AC004901.1* | 2.9 | 2.17 | 2.79 | 0.29 |
| ENSG00000112541 | *PDE10A* | 2.62 | 2.51 | 2.78 | 0.22 |
| ENSG00000187091 | *PLCD1* | 1.79 | 2.54 | 2.78 | 0.03 |
| ENSG00000259107 | *CTD-2128A3.1* | 2.82 | 0.14 | 2.76 | 0.09 |
| ENSG00000196639 | *HRH1* | 2.45 | 2.45 | 2.7 | 0.18 |
| ENSG00000135636 | *DYSF* | 2.24 | 1.17 | 2.62 | 0.09 |
| ENSG00000137573 | *SULF1* | 2.87 | 1.55 | 2.61 | 0.3 |
| ENSG00000179796 | *LRRC3B* | 2.29 | 1.62 | 2.55 | 0.2 |
| ENSG00000231703 | *RP4-669H2.1* | 1.7 | 1.64 | 2.54 | 0.07 |
| ENSG00000181982 | *CCDC149* | 1.77 | 1.93 | 2.53 | 0.14 |
| ENSG00000246223 | *C14orf64* | 2.16 | 1.21 | 2.53 | 0.03 |
| ENSG00000223414 | *LINC00473* | 2.53 | 1.42 | 2.52 | 0.08 |
| ENSG00000254946 | *RP11-531H8.1* | 1.96 | 2.47 | 2.52 | 0.14 |
| ENSG00000071073 | *MGAT4A* | 2.16 | 1.99 | 2.51 | 0.11 |
| ENSG00000226856 | *AC093901.1* | 2.7 | 1.74 | 2.48 | 0.21 |
| ENSG00000229192 | *AC004870.3* | 2.05 | 1.78 | 2.48 | 0.12 |
| ENSG00000140937 | *CDH11* | 2.34 | 1.2 | 2.44 | 0.12 |
| ENSG00000233715 | *RP4-798P15.2* | 2.82 | 0.45 | 2.43 | 0.04 |
| ENSG00000250250 | *CTD-2350J17.1* | 2.32 | 1.68 | 2.41 | 0.15 |
| ENSG00000077782 | *FGFR1* | 2.39 | 1.48 | 2.4 | 0.14 |
| ENSG00000172901 | *AQPEP* | 2.12 | 1.74 | 2.4 | 0.13 |
| ENSG00000225493 | *AC092619.1* | 2.21 | 0.46 | 2.39 | 0.02 |
| ENSG00000234273 | *AC073071.1* | 1.98 | 1.78 | 2.39 | 0.1 |
| ENSG00000055813 | *CCDC85A* | 1.95 | 1.76 | 2.38 | 0.16 |
| ENSG00000256956 | *RP11-252P19.3* | 2.09 | 1.99 | 2.35 | 0.15 |
| ENSG00000236757 | *AC007251.2* | 2.37 | 1.34 | 2.34 | 0.04 |
| ENSG00000111713 | *GYS2* | 2.21 | 1.38 | 2.33 | 0.14 |
| ENSG00000074657 | *ZNF532* | 2.18 | 1.89 | 2.3 | 0.16 |
| ENSG00000248319 | *RP11-205M3.3* | 2.28 | 1.56 | 2.3 | 0.06 |
| ENSG00000256879 | *RP11-284H19.1* | 2.36 | 0.83 | 2.3 | 0.04 |
| ENSG00000268388 | *FENDRR* | 2.39 | 0 | 2.29 | 0.05 |
| ENSG00000251615 | *RP11-774O3.3* | 2.32 | -1.13 | 2.28 | 0.03 |
| ENSG00000079102 | *RUNX1T1* | 2.03 | 1.91 | 2.26 | 0.17 |
| ENSG00000253702 | *RP11-567J20.1* | 1.89 | 2.29 | 2.24 | 0.07 |
| ENSG00000118407 | *FILIP1* | 2.27 | 1.87 | 2.23 | 0.17 |
| ENSG00000250410 | *RP11-714G18.1* | 1.73 | 2.06 | 2.22 | 0.03 |
| ENSG00000254968 | *RP11-65M17.3* | 1.75 | 1.32 | 2.2 | 0.05 |
| ENSG00000121297 | *TSHZ3* | 2.06 | 1 | 2.18 | 0.01 |
| ENSG00000196960 | *AL117340.1* | 2.26 | 1.05 | 2.17 | 0.02 |
| ENSG00000005981 | *ASB4* | 1.63 | 1.9 | 2.16 | 0.1 |
| ENSG00000178473 | *UCN3* | 2.17 | 0 | 2.15 | 0.02 |
| ENSG00000235269 | *AL162759.1* | 2.11 | 1.17 | 2.15 | 0.12 |
| ENSG00000244968 | *LIFR-AS1* | 2.14 | 1.54 | 2.15 | 0.17 |
| ENSG00000258168 | *RP11-588H23.3* | 1.7 | 1.6 | 2.15 | 0.09 |
| ENSG00000105643 | *ARRDC2* | 0 | 2.11 | 2.14 | 0.03 |
| ENSG00000133116 | *KL* | 1.84 | 1.56 | 2.13 | 0.09 |
| ENSG00000154262 | *ABCA6* | 2 | 1.37 | 2.13 | 0.03 |
| ENSG00000249631 | *RP11-281P23.2* | 1.96 | 1.71 | 2.1 | 0.1 |
| ENSG00000255240 | *RP11-142C4.6* | 2.17 | 0.76 | 2.1 | 0.1 |
| ENSG00000113811 | *SELK* | 1.86 | 1.61 | 2.07 | 0.02 |
| ENSG00000166840 | *GLYATL1* | 1 | 2.19 | 2.07 | 0.05 |
| ENSG00000236297 | *RP11-175P19.2* | 1.59 | 1.65 | 2.07 | 0.07 |
| ENSG00000260364 | *RP11-256I9.3* | 2.13 | 0.98 | 2.05 | 0.1 |
| ENSG00000152128 | *TMEM163* | 1.68 | 1.29 | 2.04 | 0.09 |
| ENSG00000233491 | *AC010091.1* | 2.01 | -0.15 | 2.04 | 0.02 |
| ENSG00000126785 | *RHOJ* | 1.73 | 1.72 | 2.03 | 0.11 |
| ENSG00000172554 | *SNTG2* | 1.97 | 1.9 | 2.01 | 0.11 |
| ENSG00000182793 | *GSTA5* | 1.62 | 1.62 | 1.99 | 0.13 |
| ENSG00000154479 | *CCDC173* | 1.68 | 1.36 | 1.97 | 0.05 |
| ENSG00000196177 | *ACADSB* | 1.68 | 1.29 | 1.97 | 0.05 |
| ENSG00000198216 | *CACNA1E* | 1.41 | 1.41 | 1.97 | 0.07 |
| ENSG00000258773 | *RP11-255M2.2* | 1.96 | 1.72 | 1.97 | 0.11 |
| ENSG00000253385 | *KB-1254G8.1* | 1.4 | 1.9 | 1.95 | 0.02 |
| ENSG00000253960 | *RP11-388K12.1* | 1.87 | 0 | 1.95 | 0.02 |
| ENSG00000256222 | *MTRNR2L3* | 0.92 | 1.87 | 1.95 | 0.03 |
| ENSG00000225179 | *LINC00457* | 1.26 | 1.41 | 1.94 | 0.06 |
| ENSG00000102468 | *HTR2A* | 1.58 | 1.74 | 1.92 | 0.06 |
| ENSG00000167191 | *GPRC5B* | 1 | 1.81 | 1.92 | 0.03 |
| ENSG00000237838 | *AC133680.1* | 1.87 | 1.09 | 1.92 | 0.05 |
| ENSG00000255005 | *RP11-90K17.2* | 2.06 | 1 | 1.92 | 0.06 |
| ENSG00000104723 | *TUSC3* | 1.66 | 1.55 | 1.91 | 0.06 |
| ENSG00000188585 | *LINC00083* | 1.88 | -0.07 | 1.9 | 0.01 |

*Note:* MDA, mean decrease accuracy; MDG, mean decrease gini.
