# Supplementary material for: 5-Hydroxymethylome in Circulating Cell-free DNA as A Potential Biomarker for Non-small-cell Lung Cancer
Source: Genomics Proteomics Bioinformatics. 2018 Jul 18;16(3):187–99. doi: 10.1016/j.gpb.2018.06.002 (PMC6076378; doi:10.1016/j.gpb.2018.06.002)
Supplement: Supplementary Table S3 [file mmc6.docx]

**Table S3 The 5hmC level of clinically known and potential biomarkers**

|  | **Known clinical markers** | | | |  | **Candidate markers** | | | | | |
| --- | --- | --- | --- | --- | --- | --- | --- | --- | --- | --- | --- |
|  | **CEA** | **CA125** | **NSE** | **CYFRA21-1** |  | ***RSPO3*** | ***LDB2*** | ***ZNF679*** | ***SIPA1L2*** | ***AP001604.3*** | ***RP1-137K24.1*** |
| **Criteria** | < 5 μg/l | < 35 U/ml | < 17 ng/ml | 0.1－3.3 ng/ml |  | FPKM < 0.1875 | FPKM < 0.3214 | FPKM < 0.0835 | FPKM < 0.5392 | FPKM < 0.1202 | FPKM < 0.1366 |
| **Lung_7** | 1.51 | 7.78 | 20.27 | 3.18 |  | 0.1984 | 0.3838 | 0.0902 | 0.5363 | 0.1595 | 0.1488 |
| **Lung_13** | 8.26 | 10.56 | 3.73 | 6.97 |  | 0.2712 | 0.6780 | 0.1035 | 0.8272 | 0.2367 | 0.1742 |
| **Lung_13** | 2.54 | 9.58 | 10.48 | 2.46 |  | 0.1580 | 0.3410 | 0.1047 | 0.6534 | 0.1272 | 0.1481 |
| **Lung_6** | 1.66 | 13.62 | 13.19 | 1.11 |  | 0.2520 | 0.7640 | 0.0884 | 0.7381 | 0.2166 | 0.1902 |
| **Lung_14** | 54.04 | 16.24 | 15.23 | 1.3 |  | 0.3036 | 0.5265 | 0.1444 | 0.6632 | 0.1748 | 0.1647 |
| **Lung_11** | 2.57 | 8.23 | 12.07 | 1.66 |  | 0.3306 | 0.7128 | 0.0990 | 0.8383 | 0.3094 | 0.2215 |
| **Lung_17** | 2.41 | 12.16 | 9.13 | 2.36 |  | 0.2139 | 0.4394 | 0.1312 | 0.7965 | 0.1753 | 0.2394 |
| **Lung_19** | 3.39 | 14.66 | 11.96 | 2.49 |  | 0.2257 | 0.4789 | 0.1346 | 0.6875 | 0.1968 | 0.1928 |
| **Lung_24** | 1.83 | 6.15 | 12.49 | 1.73 |  | 0.2230 | 0.5565 | 0.1804 | 0.7534 | 0.2564 | 0.2457 |
| **Lung_26** | 4.03 | 7.13 | 9.9 | 3.66 |  | 0.3191 | 0.6541 | 0.1392 | 0.8582 | 0.2785 | 0.2112 |
| **Lung_22** | 1.41 | 22.16 | 16.31 | 3.57 |  | 0.2435 | 0.5998 | 0.0906 | 0.7207 | 0.2274 | 0.2092 |
| **Lung_29** | 1.38 | 21.24 | 7.92 | 2.68 |  | 0.2022 | 0.4167 | 0.1212 | 0.6247 | 0.1649 | 0.1749 |
| **Lung_30** | 142 | 138.2 | 13.1 | 8.27 |  | 0.2791 | 0.7351 | 0.1232 | 0.7797 | 0.2806 | 0.1919 |
| **Lung_28** | 1.36 | 5.61 | 8.44 | 3.45 |  | 0.2383 | 0.5773 | 0.1816 | 0.7786 | 0.2427 | 0.2634 |
| **Lung_32** | 3.26 | 13.88 | 9.39 | 2.04 |  | 0.3456 | 0.7195 | 0.1067 | 0.8127 | 0.2314 | 0.3109 |
| **Lung_33** | 1.12 | 11.02 | 14.53 | 3.81 |  | 0.1726 | 0.4518 | 0.0756 | 0.5495 | 0.1924 | 0.1084 |
| **Lung_39** | 4.04 | 9.19 | 10.75 | 3.2 |  | 0.1600 | 0.2807 | 0.1082 | 0.5503 | 0.0588 | 0.0994 |
| **Lung_43** | 2.52 | 15 | 9.96 | 2.5 |  | 0.2698 | 0.5761 | 0.1510 | 0.6572 | 0.2006 | 0.2480 |
| **Lung_46** | 3.79 | 71.03 | 10.35 | 3.65 |  | 0.2208 | 0.4384 | 0.0952 | 0.6902 | 0.2252 | 0.1680 |
| **Lung_50** | 3.47 | 15.52 | 10.53 | 4.13 |  | 0.1738 | 0.2722 | 0.0998 | 0.4758 | 0.1311 | 0.1385 |
| **Lung_54** | 3.991 | 9.554 | 6.782 | 1.97 |  | 0.4361 | 0.7919 | 0.1598 | 0.8766 | 0.3092 | 0.3333 |
| **Lung_64** | 2.54 | 27.61 | 12.75 | 1.53 |  | 0.1921 | 0.3926 | 0.0739 | 0.5065 | 0.1555 | 0.1555 |
| **Lung_65** | 1.299 | 2.016 | 7.595 | 1.91 |  | 0.3335 | 0.7807 | 0.1853 | 0.7641 | 0.2995 | 0.2923 |
